# Plasma Eicosanoid Levels in Tuberculosis and Tuberculosis-Diabetes Co-morbidity Are Associated With Lung Pathology and Bacterial Burden

**DOI:** 10.3389/fcimb.2019.00335

**Published:** 2019-10-01

**Authors:** Nathella Pavan Kumar, Kadar Moideen, Arul Nancy, Vijay Viswanathan, Basavaradhya S. Shruthi, Sivakumar Shanmugam, Syed Hissar, Hardy Kornfeld, Subash Babu

**Affiliations:** ^1^National Institutes of Health—NIRT—International Center for Excellence in Research, Chennai, India; ^2^Prof. M. Viswanathan Diabetes Research Center, Chennai, India; ^3^Department of Bacteriology, National Institute for Research in Tuberculosis, Chennai, India; ^4^Department of Clinical Research, National Institute for Research in Tuberculosis, Chennai, India; ^5^University of Massachusetts Medical School, Worcester, MA, United States; ^6^Laboratory of Parasitic Diseases, National Institutes of Allergy and Infectious Diseases, National Institutes of Health, Bethesda, MD, United States

**Keywords:** *Mycobacterium tuberculosis*, diabetes mellitus, eicosanoids, anti-TB treatment, cytokines

## Abstract

Host eicosanoids are lipid mediators of inflammation that are commonly accepted as important modulators of the host immune response in *Mycobacterium tuberculosis* infection. During active tuberculosis (TB), eicosanoids may play an important role in the regulation of inflammatory responses. However, a detailed investigation of the relationship of eicosanoids in TB and TB-diabetes comorbidity (TB-DM) and association to disease pathology or bacterial burdens has not been studied. To study this, we examined the plasma levels of Lipoxin A4 (LXA4), 15-epi-LXA4, Leukotriene B4 (LTB4), and Prostaglandin E2 (PGE2) in individuals with either TB-DM, TB, diabetes mellitus (DM) or healthy controls (HC). Plasma levels of LXA4, 15-epi-LXA4, and PGE2 were significantly increased while the levels of LTB4 were significantly decreased in TB-DM and TB group compared to DM and HC. The ratio of LXA4 to LTB4 and 15-epiLXA4 to LTB4 was significantly enhanced in TB-DM compared to TB. Moreover, the levels of LXA4, 15-epi-LXA4 and the ratios of LXA4 to LTB4 and 15-epiLX4 to LTB4 were significantly increased in TB individuals with bilateral or cavitary disease and these markers also revealed a significant positive relationship with bacterial burden. At the completion of anti-tuberculosis therapy (ATT), levels of LXA4, 15-epi-LXA4, and PGE2 in TB-DM and TB groups were diminished and levels of LTB4 were enhanced in the TB group compared to pre-treatment. Our data imply that alteration and upregulation of eicosanoids are standard characteristics of TB-DM co-morbidity. Our data also demonstrate that modulation in the eicosanoid levels reflect disease severity and extent in TB and TB-DM and are modulated by ATT.

## Introduction

Tuberculosis (TB) and diabetes mellitus (DM) are two of the most common diseases worldwide and often occur in the same geographical regions (Dooley and Chaisson, 2009). The occurrence of this co-morbidity poses a major threat to the global program for elimination of tuberculosis (Lonnroth et al., 2015). While a variety of clinical and epidemiological studies have been performed in this dual disease process, very few immunological or translational insights are available. The presence of a hyper-inflammatory milieu is highly characteristic of TB-DM co-morbidity and provides an opportunity for host-directed therapies to function as an adjunct measure to control this threat (Prada-Medina et al., 2017).

Eicosanoids are arachidonic acid derived lipid mediators that elicit a panel of pro- and anti-inflammatory responses and include prostaglandins, lipoxins, leukotrienes, and resolvins (Tobin et al., 2013). The enzyme 5-lipoxygenase is essential for the generation of lipoxin and leukotriene mediators from arachidonic acid, while the enzymes cyclooxygenase−1 and 2 are required for the generation of prostaglandins (Das, 2017). These lipid mediators have been shown to exert major influence on the outcomes of experimental *M. tuberculosis* (*M. tb)* infection (Mayer-Barber and Sher, 2015). Published studies have reported that leukotriene B4 (LTB4) and prostaglandin E2 (PGE2) play a host protective role by mediating bacterial clearance, while lipoxin A4 (LXA4) and 15-epi-lipoxin A4 (15-epi-LXA4) play a pathogenic role by hampering the host inflammatory response in TB (Mayer-Barber et al., 2014). Moreover, pro- and anti-inflammatory eicosanoid ratios are associated with the modulation of the host response to *M. tb* infection (Bafica et al., 2005; Tobin et al., 2012). Published studies have also reported that eicosanoid ratios in plasma were significantly increased in active TB patients compared to latent TB or healthy controls (Mayer-Barber et al., 2014). However, the role in eicosanoids in human TB and more specifically TB-DM co-morbidity has been poorly explored.

In this study, we elucidated the systemic levels of eicosanoids at baseline and at the end of anti-tuberculosis treatment (ATT). Our data reveal that DM differentially modulates the eicosanoid levels in individuals with TB before and after completion of treatment. Our data also show that certain eicosanoid levels reflect baseline disease severity and extent in TB and TB-DM and are modulated by ATT.

## Materials and Methods

### Ethics Statement

The Ethics Committees of the Prof. M. Viswanathan Diabetes Research Center and National Institute for Research in Tuberculosis provided approval for this study. Informed written consent was obtained from all individuals recruited for the study.

### Study Population

Plasma samples were collected from 44 participants with active pulmonary TB with diabetes mellitus (TB-DM) and 44 participants with active pulmonary TB (TB), 44 participants with diabetes mellitus (DM), 30 healthy control participants with no TB or diabetes (HC) recruited in Chennai, India. Pulmonary TB was diagnosed based on smear and culture positivity for *M. tb*. Chest X-rays were used to define cavitary disease (*n* = 23) and non-cavitary disease (*n* = 65) as well as unilateral (*n* = 49) vs. bilateral (*n* = 39) lung involvement. Smear grades were used to estimate bacterial burdens and classified as 1+ (*n* = 33), 2+ (*n* = 33) and 3+ (*n* = 22). All participants with active TB had no record of prior TB disease or ATT at the time of enrolment. Oral glucose tolerance test and/or glycated hemoglobin (HbA1c) levels (for known diabetics) was used to diagnose glycemic status (DM or normoglycemia), according to the WHO criteria. All the enrolled DM and HC participants were Quantiferon TB gold assay negative, asymptomatic and with normal chest X-rays. Standard ATT was administered to TB-DM and TB participants using the directly observed treatment, short course (DOTS) strategy. Fresh plasma samples were obtained again from TB-DM and TB participants at the end of ATT (6 months). All TB-DM and TB participants were culture negative for *M. tb* at this time point.

### ELISA

Plasma levels of LXA4, 15-epi-LXA4, PGE2, and LTB4 were measured using the MyBioSource.com quantitative measurement kit and plasma levels of IL-1α, IL-1β, IFNγ, and TNFα were measured using Bio-Plex multiplex cytokine assay system (Bio-Rad, Hercules, CA). The lowest detection limits were as follows LXA4, 0.156 ng/mL; 15-epi-LXA4, 0.312 ng/mL; PGE2, 7.8 pg/mL; LTB4, 15.6 pg/mL; IL-1α, 5.23 pg/mL; IL-1β, 3.96 pg/mL; IFNγ, 4.39 pg/mL, and TNFα, 3.24 pg/mL.

### Statistical Analysis

Geometric means (GM) were used for measurements of central tendency. Statistically significant differences between the four groups were analyzed using the Kruskal-Wallis test with Dunn's correction for multiple comparisons. The Mann-Whitney test was used to compare eicosanoid concentrations in TB individuals with unilateral or bilateral lung lesions and cavitary or non-cavitary disease. Linear trend post-test was used to compare eicosanoid concentrations with smear grades (reflecting bacterial burdens) and Spearman rank correlation was used to compare eicosanoid concentrations with HbA1c levels. Analyses were performed using GraphPad PRISM Version 8.

## Results

### Study Population Characteristics

Table 1 depicts the demographic and biochemical features of the study population. As shown, the TB-DM and TB groups did not differ significantly in age, sex, smear, or culture grades at baseline (Table 1).

**Table 1:** Demographic and clinical variables of the study groups and biochemical parameters in TB-DM, TB, DM, and HC.

**Study demographics**	**TB-DM**	**TB**	**DM**	**HC**
No. of subjects recruited	44	44	44	30
Gender (male/female)	34/10	27/17	30/14	15/15
Median age (range)	47 (25–70)	39 (24–67)	44 (33–68)	34 (23–55)
Smear grade: 0/1+/2+/3+	0/14/19/11	0/19/18/7	NA	NA
Cavitary disease (Y/N)	13/31	10/34	NA	NA
Lung lesions (unilateral/bilateral)	24/20	25/19	NA	NA
Fasting blood glucose, mg/dL	154 (111–417)	93 (73–103)	140 (95–311)	75 (70–109)
Glycated hemoglobin level, %	10.3 (7.3–15.6)	5.6 (5.0–5.8)	10 (6.9–12.5)	5.5 (5.0–5.9)

*The values represent the geometric mean (and the 95% confidence intervals) except for age where the median (and the range) are depicted*.

### Plasma Levels of Eicosanoids and Ratios in TB and TB-DM

**Figure 1:**
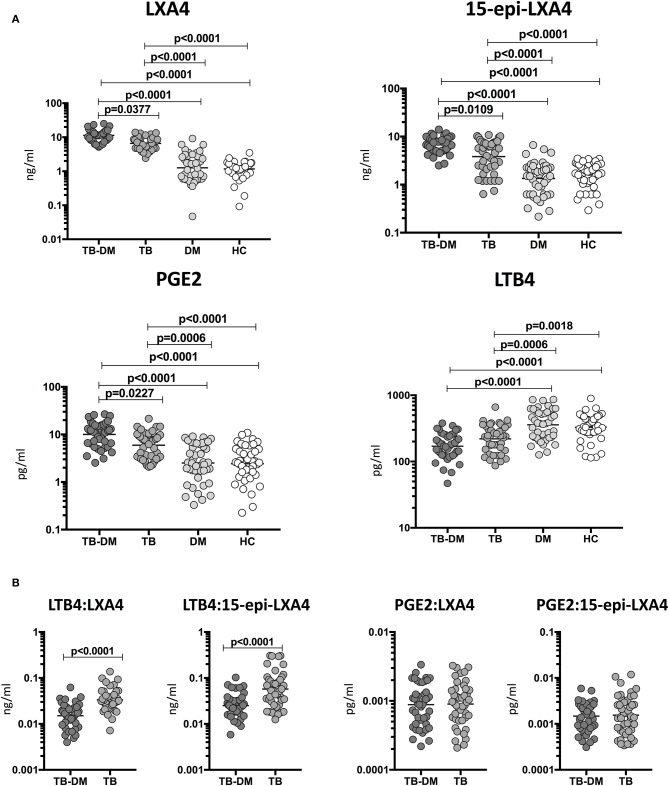
Altered plasma levels of eicosanoids in TB-DM and TB individuals. **(A)** The plasma levels of LXA4, 15-epi-LXA4, PGE2, and LTB4 were measured in TB-DM (*n* = 44), TB (*n* = 44), DM (*n* = 44), and HC (*n* = 44) individuals at baseline. **(B)** Ratios of LXA4:LTB4, 15-epi-LXA4:LTB4, LXA4:PGE2, 15-epi-LXA4:PGE2, and PGE2:LTB4 in TB-DM and TB individuals at baseline. The data are represented as scatter plots with each circle representing a single individual. **(A)** *P*-values were calculated using the Kruskal-Wallis test with Dunn's *post-hoc* for multiple comparisons. **(B)** *P*-values were calculated using Mann-Whitney test.

To determine the impact of TB and DM on eicosanoid expression, we measured the plasma levels of LXA4, 15-epiLXA4, PGE2, and LTB4 in TB-DM, TB, DM, and HC participants (Figure 1A). The levels of LXA4 (Geometric Mean (GM) 11.3 ng/ml in TB-DM vs. 6.6 ng/ml in TB, 1.3 pg/ml in DM and 2.04 pg/ml in HC), 15-epiLXA4 (GM 6.8 ng/ml in TB-DM vs. 3.9 ng/ml in TB, 1.4 ng/ml in DM and 1.7 ng/ml in HC), and PGE2 (GM 10 pg/ml in TB-DM vs. 5.9 pg/ml in TB, 2.5 pg/ml in DM and 2.4 pg/ml in HC) were significantly elevated in TB-DM and DM group in comparison with TB and HC group. In contrast, the levels of LTB4 (GM 170.3 pg/ml in TB-DM vs. 220.3 pg/ml in TB, 357.8 pg/ml in DM and 329.8 pg/ml in HC) was significantly decreased in TB-DM and DM group in comparison with TB and HC individuals. Thus, TB-DM and TB were associated with significantly altered plasma levels of eicosanoids. Next, to examine the association of eicosanoid ratios with TB-DM and TB, we measured the ratios of LXA4:LTB4, 15-epi-LXA4:LTB4, LXA4:PGE2, 15-epi-LXA4:PGE2, and PGE2:LTB4 in TB-DM and TB individuals (Figure 1B). The ratios of LXA4:LTB4 (GM 0.06643 in TB-DM vs. 0.02994 in TB), 15-epi-LXA4:LTB4 (GM 0.03992 in TB-DM vs. 0.01744 in TB), and PGE2:LTB4 (GM 0.05873 in TB-DM vs. 0.02691 in TB) were significantly increased in TB-DM compared to TB. Thus, balance between lipid mediators is associated with differences in TB and TB-DM.

### LXA4 and 15-epiLXA4 Are Markers of Disease Severity in TB and TB-DM

**Figure 2:**
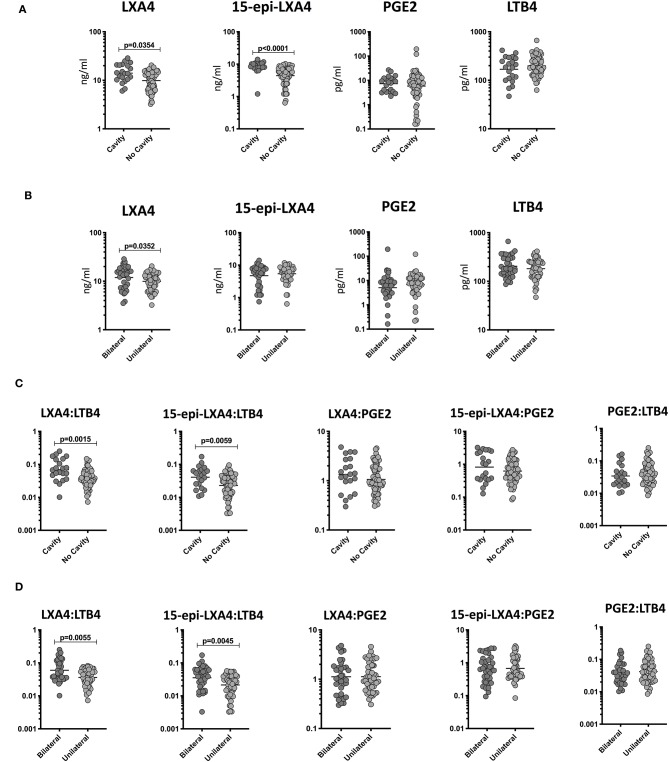
Elevated plasma levels of eicosanoids in cavitary and bilateral disease and relationship to bacterial burden in TB-DM and TB individuals. **(A)** The plasma levels of LXA4, 15-epi-LXA4, PGE2, and LTB4 were measured in TB-DM and TB individuals with cavitary vs. non-cavitary disease. **(B)** The plasma levels of LXA4, 15-epi-LXA4, PGE2, and LTB4 were measured in TB-DM and TB individuals with bilateral vs. unilateral disease. **(C)** The ratios of LXA4:LTB4, 15-epi-LXA4:LTB4, LXA4:PGE2, 15-epi-LXA4:PGE2, and PGE2:LTB4 were measured in TB-DM and TB individuals with cavitary vs. non-cavitary disease **(D)** The ratios of LXA4:LTB4, 15-epi-LXA4:LTB4, LXA4:PGE2, 15-epi-LXA4:PGE2, and PGE2:LTB4 were measured in TB-DM and TB individuals with bilateral vs. unilateral disease. The data are represented as scatter plots with each circle representing a single individual. *P*-values were calculated using the Mann-Whitney test with Holm's correction for multiple comparisons.

To determine the relationship among the plasma levels of eicosanoids and disease severity in TB and TB-DM, we compared plasma levels of LXA4, 15-epiLXA4, PGE2, and LTB4 in all TB study participants with unilateral vs. bilateral disease and cavitary vs. non-cavitary disease based on chest X-ray. As shown in Figure 2A, the plasma levels of LXA4 (GM 14 ng/ml in cavitary vs. 9 ng/ml in non-cavitary disease) and 15-epiLXA4 (GM 8.2 ng/ml in cavitary vs. 4.4 ng/ml in non-cavitary) were significantly elevated in TB-DM and TB participants with cavitary disease compared to those without. Similarly, as shown in Figure 2B, the plasma levels of LXA4 (GM 12 ng/ml in bilateral vs. 9 ng/ml in unilateral disease) were significantly elevated in TB-DM participants with bilateral disease compared to those with unilateral disease. To elucidate the relationship between the ratios of eicosanoids and disease severity in TB and TB-DM, we compared ratios of LXA4:LTB4, 15-epi-LXA4:LTB4, LXA4:PGE2, 15-epi-LXA4:PGE2, and PGE2:LTB4 in all TB study participants with unilateral vs. bilateral disease and cavitary vs. non-cavitary disease based on chest X-ray. As shown in Figure 2C, the ratios of LXA4:LTB4 (GM 0.06902 in cavitary vs. 0.0303550 in non-cavitary disease) and 15-epi-LXA4:LTB4 (GM 0.04095 in cavitary vs. 0.02299 in non-cavitary) were significantly elevated in TB-DM and TB participants with cavitary disease compared to those without. Similarly, as shown in Figure 2D, the ratios of LXA4:LTB4 (GM 0.05959 in bilateral vs. 0.03628 in unilateral disease) and 15-epi-LXA4:LTB4 (GM 0.03531 in bilateral vs. 0.02140 in unilateral disease) were significantly elevated in TB-DM and TB participants with bilateral disease compared to those with unilateral disease. Thus, disease severity assessed radiographically in TB-DM and TB was associated with elevated plasma levels of certain eicosanoids, most notably LXA4 and altered ratios of LXA4 or epi-LXA4 to LTB4.

### LXA4 and 15-epiLXA4 Are Markers of Bacterial Burden in TB and TB-DM

**Figure 3:**
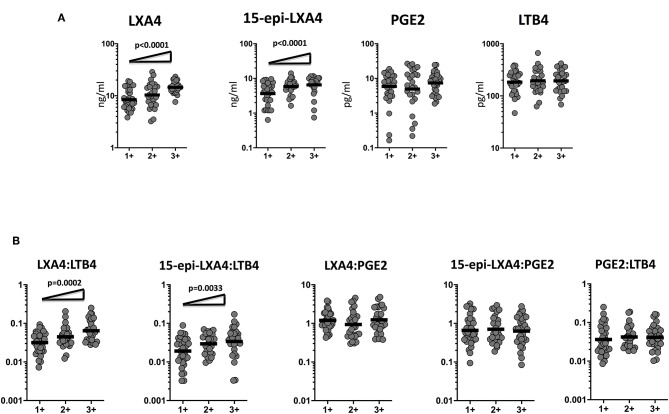
Eicosanoid are markers of bacterial burden in TB and TB-DM. **(A)** The relationship between the plasma levels of LXA4, 15-epi-LXA4, PGE2, and LTB4 and smear grades as estimated by sputum smears was examined in TB-DM and TB individuals. **(B)** The relationship between the ratios of LXA4:LTB4, 15-epi-LXA4:LTB4, LXA4:PGE2, 15-epi-LXA4:PGE2, and PGE2:LTB4 and smear grades as estimated by sputum smears was examined in TB-DM and TB individuals. The data are represented as scatter plots with each circle representing a single individual. For bacterial burden correlation *P*-values were calculated using the Linear trend post-test.

To determine the association of the plasma levels of eicosanoids and bacterial burdens, we studied a correlation of the plasma levels of LXA4, 15-epiLXA4, PGE2, and LTB4 in TB-DM and TB study participants with smear grades from 1+ to 3+. As shown in Figure 3A, LXA4 and 15-epiLXA4 showed a significant positive correlation with smear grades in TB-DM and TB participants, indicating a positive relationship of these factors with bacterial burdens. To elucidate the relationship of the ratios of eicosanoids and bacterial burdens, we studied the correlation of the ratios of LXA4:LTB4, 15-epi-LXA4:LTB4, LXA4:PGE2, 15-epi-LXA4:PGE2, and PGE2:LTB4 in TB-DM and TB participants with smear grades. As shown in Figure 3B, LXA4:LTB4 and 15-epi-LXA4:LTB4 showed a significant positive relationship with smear grades in TB-DM and TB participants, demonstrating a positive association of these factors with bacterial burdens. Thus, disease severity by estimated bacterial burden in TB-DM and TB was associated with increased systemic levels of certain eicosanoids, most notably LXA4 and altered ratios of LXA4 or epi-LXA4 to LTB4.

### Decreased Plasma Levels of Eicosanoids in TB-DM Following ATT

**Figure 4:**
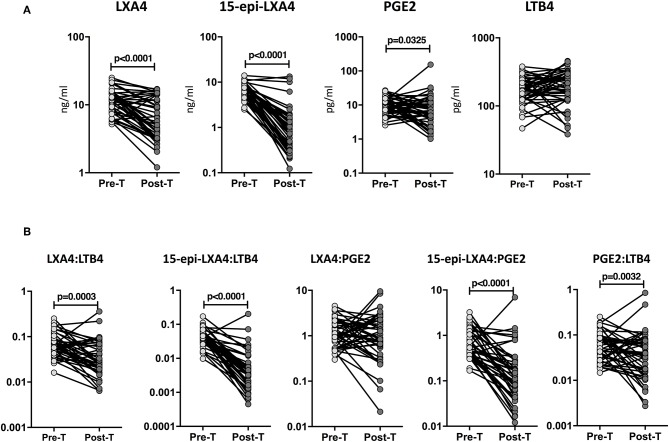
Altered plasma levels of eicosanoids at the end of standard anti-tuberculosis therapy in TB-DM individuals. **(A)** The plasma levels of LXA4, 15-epi-LXA4, PGE2, and LTB4 were measured in TB-DM individuals at baseline (pre-T) and at 6 months of ATT (post-T). **(B)** The ratios of LXA4:LTB4, 15-epi-LXA4:LTB4, LXA4:PGE2, 15-epi-LXA4:PGE2, and PGE2:LTB4 were measured in TB-DM individuals at baseline (pre-T) and at 6 months of ATT (post-T). The data are presented as line graphs with each line representing a single individual. *P*-values were calculated using the Wilcoxon signed rank test.

To determine whether the eicosanoid levels were altered by ATT, we examined the plasma levels of eicosanoids in TB-DM at baseline (pre-T) and at the completion of ATT (post-T). As shown in Figure 4A, ATT resulted in significantly decreased levels of LXA4 (GM of 11.31 ng/ml at pre-T vs. 6.1 ng/ml at post-T), 15-epiLXA4 (GM of 6.7 ng/ml at pre-T vs. 1.2 ng/ml at post-T) and PGE2 (GM of 10 pg/ml at pre-T vs. 6.3 pg/ml at post-T) in TB-DM individuals. To elucidate whether the eicosanoid ratios were also modulated by ATT, we examined the eicosanoid ratios in TB-DM and TB at baseline (pre-T) and at the completion of ATT (post-T). As shown in Figure 4B, ATT resulted in significantly decreased ratios of LXA4:LTB4 (GM of 0.06643 at pre-T vs. 0.03306 at post-T), 15-epi-LXA4:LTB4 (GM of 0.03992 at pre-T vs. 0.004554 at post-T), 15-epi-LXA4:PGE2 (GM of 0.6798 at pre-T vs. 0.1326 at post-T), and PGE2:LTB4 (GM of 0.05873 at pre-T vs. 0.03434 at post-T) in TB-DM individuals. Thus, eicosanoid levels and eicosanoid ratios are significantly altered in TB-DM by ATT.

### Decreased Plasma Levels of Eicosanoids in TB Following ATT

**Figure 5:**
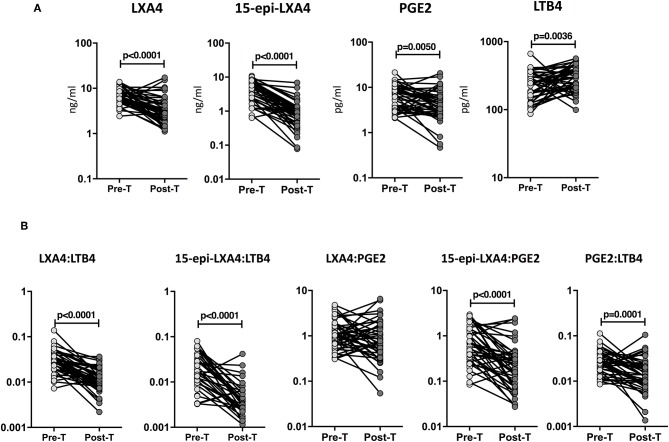
Increased eicosanoids ratios at the end of standard anti-tuberculosis therapy in TB-DM and TB individuals. **(A)** The plasma levels of LXA4, 15-epi-LXA4, PGE2, and LTB4 were measured in TB individuals at baseline (pre-T) and at 6 months of ATT (post-T). **(B)** The ratios of LXA4:LTB4, 15-epi-LXA4:LTB4, LXA4:PGE2, 15-epi-LXA4:PGE2, and PGE2:LTB4 were measured in TB individuals at baseline (pre-T) and at 6 months of ATT (post-T). The data are presented as line graphs with each line representing a single individual. *P*-values were calculated using the Wilcoxon signed rank test.

To examine whether the eicosanoid levels were modulated by ATT, we studied the eicosanoid ratios in TB at baseline (pre-T) and at the completion of ATT (post-T). As shown in Figure 5A, ATT resulted in significantly decreased levels of LXA4 (GM of 6.5 ng/ml at pre-T vs. 3.1 ng/ml at post-T), 15-epiLXA4 (GM of 3.8 ng/ml at pre-T vs. 0.77 ng/ml at post-T), and PGE2 (GM of 5.9 pg/ml at pre-T vs. 3.5 pg/ml at post-T) and significantly increased levels of LTB4 (GM of 220.3 pg/ml at pre-T vs. 283.8 pg/ml at post-T) in TB individuals. Similarly, as shown in Figure 5B, ATT resulted in significantly decreased levels of LXA4:LTB4 (GM of 0.02994 at pre-T vs. 0.01124 at post-T), 15-epi-LXA4:LTB4 (GM of 0.01744 at pre-T vs. 0.002713 at post-T), 15-epi-LXA4:PGE2 (GM of 0.6479 at pre-T vs. 0.1883 at post-T), and PGE2:LTB4 (GM of 0.02691 at pre-T vs. 0.01441 at post-T) in TB individuals. Thus, eicosanoid levels and eicosanoid ratios are significantly altered in TB by ATT.

### Circulating Eicosanoids Exhibit Relationship With Pro-Inflammatory Cytokines

**Figure 6:**
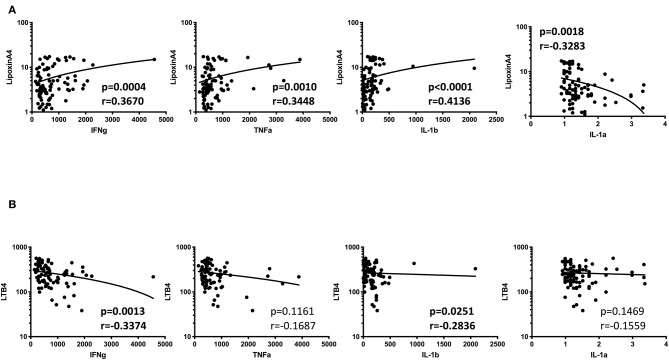
Significant correlation between plasma levels of eicosanoids and pro-inflammatory cytokines in all TB individuals. **(A)** The relationship between the plasma levels of lipoxin A4 and cytokines was examined in all TB individuals with and without DM at baseline. **(B)** The relationship between the plasma levels of LTB4 and cytokines was examined in all TB individuals with and without DM at baseline The data are represented as scatter plots with each circle representing a single individual. *P*-values were calculated using the Spearman Rank Correlation.

We have previously measured the circulating levels of pro-inflammatory cytokines (IL-1α, IL-1β, IFNγ, and TNFα) in these individuals and shown that IL-1β, IFNγ, and TNFα were significantly increased in TB patients with DM compared to TB individuals (Kumar et al., 2013; Prada-Medina et al., 2017). Since, LXA4, LTB4, and PGE2 have been described to be associated with pro-inflammatory cytokine modulation in TB, we examined the association between the plasma levels of eicosanoids in all TB individuals with pro-inflammatory cytokines (Figure 6). As shown, the circulating levels of LXA4 showed a significant positive correlation, with IFNγ, TNFα IL-1β and negative correlation with IL-1α levels. In addition, LTB4 exhibited a significant negative relationship with IFNγ and IL-1β in all TB participants with and without DM at baseline, indicating a significant involvement of these factors with cytokines.

## Discussion

Eicosanoids and prostaglandins are major lipid moieties of considerable interest in metabolic biology. However, it is being gradually accepted that these lipid molecules have the capability to shape the immune response to infectious pathogens. Lipid mediators of the eicosanoid family have been proven to have influence on the outcome of the *M. tb* infection (Mayer-Barber and Sher, 2015). Previously published studies have also reveal that *M. tb* can also alter the host eicosanoid metabolism as a survival strategy (Divangahi et al., 2010). The interplay among the eicosanoids, PGE2 and LXA4 is known to affect the type of cell death in infected macrophages. LXA4 enhances macrophage necrosis, resulting in cell death and escape of *M. tb* to the extracellular milieu. PGE2 stimulates macrophage apoptosis, resulting in membrane integrity, bacillary containment and heightened immunity (Chen et al., 2008). LTB4, via the regulation of TNFα production (Tobin et al., 2012), can enhance attraction of neutrophils (Lammermann et al., 2013) and lead to macrophage necrosis (Tobin et al., 2012). Our findings clearly demonstrate the presence of elevated levels of LXA4, 15-epiLXA4, and PGE2 in TB-DM and TB at baseline, with only LTB4 being present at lower levels compared to controls. Our data also demonstrate a positive relationship between LXA4 and pro-inflammatory cytokines and a negative relationship between LTB4 and pro-inflammatory cytokines, suggesting a cross-regulation of eicosanoids with cytokine levels in TB.

Published findings report that high LXA4 favors the mycobacterial infection (Amaral et al., 2013). Specifically, LXA4 can induce necrotic cell death of the infected macrophages (Bafica et al., 2005). We have previously reported that LXA4 and 15-epi-LXA4 are significantly increased in the active TB group compered to latent and healthy controls (Mayer-Barber et al., 2014). In agreement with this report, our present finding also confirms the presence of elevated levels of LXA4 and 15-epi-LXA4 in TB individuals who are with and without diabetes compared to DM and HC. More interestingly, our data also highlight an important involvement of LXA4 and 15-epi-LXA4 with both the amount of pathology in TB as well as the degree of disease. Finally, our data disclose a direct correlation of LXA4 and 15-epi-LXA4 levels with bacterial burdens, perhaps suggesting that high LXA4 and 15-epi-LXA4 reflect an inflammatory environment favorable for *M. tb* replication. This is further corroborated by the fact that LXA4 and 15-epi-LXA4 levels are significantly reduced following successful chemotherapy. Interestingly, our data also reveal that only LXA4 and not 15-epiLXA exhibits a significant positive relationship with pro-inflammatory cytokines—IL-1β, IFNγ, and TNFα. Thus, LXA4 in TB-DM and TB is a major biomarker of disease severity and bacterial burden.

Earlier studies have reported that excess LTB4 production leads to increased TNF dependent macrophage cell death, while diminished levels LTB4 leads to a relative increase in LXA4, which in turn results in cell death due to loss of bacterial control (Tobin et al., 2012). Our current finding also reveals the presence of diminished levels of LTB4 in TB individuals who are with and without diabetes compared to DM and HC. Moreover, our findings reveal certain important features of eicosanoid imbalance and its association with TB-DM and DM. Thus, the ratios of LXA4: LTB4 and 15epiLXA4: LTB4 are significantly increased in TB-DM compared to TB alone. Our data also reveal that the ratio of LXA4 to LTB4 levels and of 15-epiLXA4 to LTB4 levels appear to be associated with both disease severity/extent as well as bacterial burdens in TB-DM and TB. Hence, eicosanoid balance between the lipoxins and LTB4 seems to be associative factors with lung pathology and bacterial burden in TB-DM and TB. This is further corroborated by the decrease in the ratios following ATT in both TB-DM and TB. Finally, LTB4 levels in TB-DM and TB individuals is negatively associated with the levels of the pro-inflammatory cytokines—IL-1α and IL-1β.

Animal model studies have reported PGE2 confers resistance against *M. tb* infection, while LXA4 promotes bacterial growth (Bafica et al., 2005; Chen et al., 2008; Divangahi et al., 2009). Thus, during *M. tb* infection, the balance of PGE2 and LXA4 is important in regulating the relative amounts of apoptosis and necrosis, which is an important feature in the control of intracellular infection. Moreover, studies using the genetic analysis of the susceptibility of zebrafish to *M. marinum* have validated the important role of these host lipid pathways in innate immunity (Tobin et al., 2010). Our data clearly shows that the presence of elevated levels of PGE2 are observed in TB individuals who are with and without diabetes compared to DM and HC, but our data do not reveal a direct correlation of PGE2 with lung pathology or bacterial burdens. However, we do observe significantly decrease levels of PGE2 following ATT in both TB-DM and TB individuals, suggesting that PGE2 is an important factor in TB disease. Our data also reveal no association of lipoxin to PGE2 or PGE2 to LTB4 ratios in the pathology of TB-DM and TB. Thus, PGE2 appears to play a less important associative role with disease pathology or bacterial burden in TB-DM and TB. Moreover, our data did not reveal any significant association between PGE2 levels and pro-inflammatory cytokines in TB or TB-DM.

There is an increasing appreciation of the distinct roles that lipid mediators play in regulating inflammatory responses during *M. tb* infection (Tobin and Ramakrishnan, 2013). To define the outcome and severity of mycobacterial infection, the balance between the lipid mediators are important (Pedruzzi et al., 2016). In this study, we report that ratios of LXA4:LTB4, 15-epi-LXA4:LTB4, and PGE2:LTB4 were significantly enhanced in TB-DM and correlated with disease severity and in-turn diminished following ATT. The limitations of our study are the limited sample size, the estimation of eicosanoid levels only in the plasma and the absence of mechanistic insights. Despite this, our study contributes to the growing body of literature on the regulation of innate immune responses in TB-DM co-morbidity. To our knowledge, no studies have reported correlation of LXA4:LTB4 and 15-epi-LXA4:LTB4 with disease severity in TB-DM comorbidity. Our data clearly reveals that changes in eicosanoids might reflect a perfectly balanced host response needed to control imbalance in TB diabetes comorbidity linked with outcome of *M. tb* infection. Finally, our data also complements the growing understanding of pathophysiology in TB diabetes comorbidity.

## Data Availability Statement

The datasets analyzed in this manuscript are not publicly available. Requests to access the datasets should be directed to pavankumarn@nirt.res.in.

## Ethics Statement

The studies involving human participants were reviewed and approved by Ethics Committees of the Prof. M. Viswanathan Diabetes Research Center and National Institute for Research in Tuberculosis. The patients/participants provided their written informed consent to participate in this study.

## Author Contributions

SB and NP designed the study. NP, KM, and AN conducted experiments. NP and KM acquired data. NP, KM, and SS analyzed data. VV, HK, and SB funding acquisition. BS and SH project administration. NP and SB wrote the manuscript.

### Conflict of Interest

The authors declare that the research was conducted in the absence of any commercial or financial relationships that could be construed as a potential conflict of interest.
